# Recent Advances in the Development of Fire-Resistant Biocomposites—A Review

**DOI:** 10.3390/polym14030362

**Published:** 2022-01-18

**Authors:** Elvara Windra Madyaratri, Muhammad Rasyidur Ridho, Manggar Arum Aristri, Muhammad Adly Rahandi Lubis, Apri Heri Iswanto, Deded Sarip Nawawi, Petar Antov, Lubos Kristak, Andrea Majlingová, Widya Fatriasari

**Affiliations:** 1 Department of Forest Products, Faculty of Forestry and Environment, IPB University, Bogor 16680, Indonesia; elvarawindra@yahoo.com (E.W.M.); rasyidmuhammad0505@gmail.com (M.R.R.); arumaristri@gmail.com (M.A.A.); 2 Research Center for Biomaterials BRIN, Jl Raya Bogor KM 46, Cibinong 16911, Indonesia; marl@biomaterial.lipi.go.id; 3 Department of Forest Product, Faculty of Forestry, Universitas Sumatera Utara, Medan 20155, Indonesia; 4 JATI-Sumatran Forestry Analysis Study Center, Jl. Tridharma Ujung No. 1, Kampus USU, Medan 20155, Indonesia; 5 Faculty of Forest Industry, University of Forestry, 1797 Sofia, Bulgaria; p.antov@ltu.bg; 6 Faculty of Wood Sciences and Technology, Technical University in Zvolen, 96001 Zvolen, Slovakia; kristak@tuzvo.sk (L.K.); majlingova@tuzvo.sk (A.M.)

**Keywords:** advanced biocomposites, biopolymers, natural fibers, green flame retardants, fire retardancy, product safety

## Abstract

Biocomposites reinforced with natural fibers represent an eco-friendly and inexpensive alternative to conventional petroleum-based materials and have been increasingly utilized in a wide variety of industrial applications due to their numerous advantages, such as their good mechanical properties, low production costs, renewability, and biodegradability. However, these engineered composite materials have inherent downsides, such as their increased flammability when subjected to heat flux or flame initiators, which can limit their range of applications. As a result, certain attempts are still being made to reduce the flammability of biocomposites. The combustion of biobased composites can potentially create life-threatening conditions in buildings, resulting in substantial human and material losses. Additives known as flame-retardants (FRs) have been commonly used to improve the fire protection of wood and biocomposite materials, textiles, and other fields for the purpose of widening their application areas. At present, this practice is very common in the construction sector due to stringent fire safety regulations on residential and public buildings. The aim of this study was to present and discuss recent advances in the development of fire-resistant biocomposites. The flammability of wood and natural fibers as material resources to produce biocomposites was researched to build a holistic picture. Furthermore, the potential of lignin as an eco-friendly and low-cost FR additive to produce high-performance biocomposites with improved technological and fire properties was also discussed in detail. The development of sustainable FR systems, based on renewable raw materials, represents a viable and promising approach to manufacturing biocomposites with improved fire resistance, lower environmental footprint, and enhanced health and safety performance.

## 1. Introduction

Increased environmental awareness and the scarcity of natural resources, as well as recent stringent environmental regulations and the unsustainable consumption of fossil-derived resources, have forced many manufacturing industries to search for new eco-friendly materials from renewable feedstocks to substitute conventional materials in several end uses. This growing need for “green” materials has led to the increased utilization of natural fibers in the production of biobased composite materials. The development of high-performance biocomposites fabricated from natural resources is increasing worldwide, and the greatest challenge in working with natural biobased composites is the large variations in their properties and characteristics [1] and the resulting variations in final composites. Natural fiber-reinforced composites are innovative composite materials consisting of a polymer matrix reinforced with high-strength natural fibers. The matrices currently used in the development of biobased composites are petroleum-based (thermoplastics or thermosets) or biobased (polylactide acid, polyhydroxybutyrate, starch, etc.) [2]. Thermoplastics include polymers such as polypropylene, polyethylene, polystyrene, and polyvinyl chloride, while polyesters, epoxy and polyurethane represent examples of thermosets used for manufacturing natural fiber reinforced composites. Wood, woody, and non-woody lignocellulosic biomass, all of which are renewable and sustainable materials, can be used as natural resources in biocomposites and have gained great attention in many value-added applications due to their excellent properties, such as their low cost, flexibility during processing, and highly specific stiffness, etc. [3,4,5]. Green biocomposites can be viable alternatives to the conventional synthetic fiber-reinforced composites as structural or semi-structural components, especially in lightweight applications [6,7,8]. Some of the most common applications of biobased composites include automotive panels and upholstery, window and door frames, furniture, railroad sleepers, packaging, and other applications that do not require very high mechanical properties but significantly reduce production and maintenance costs [9,10]. Biobased composite materials in the form of panels and sandwich structures have been used to replace wooden furniture, fittings, and noise-insulating panels [11]. When woody or non-woody fibers are combined with thermoplastic matrices, such as polyethylene, polypropylene, or polyvinyl chloride, wood plastic composites (WPC) are produced. Due to their excellent properties, such as high strength, durability, stiffness, and resistance to wear, these engineered materials have found a wide range of applications [12]. Despite the numerous advantages of natural fibers, there are also some drawbacks limiting their potential as a natural feedstock for the development of biobased composites, such as their insufficient adhesion and incompatibility with the matrices, lower water and thermal resistance, and their susceptibility to insect and fungi attacks, etc. In addition, they belong to the group of highly-combustible polymer materials [13]. To inhibit or suppress the burning process, refractory additives function by chemically or physically inhibiting particular stages of the burning process and lowering the amount of heat emitted during the early stages of fire by slowing its spread [14].

Although complete fire protection of biocomposites for indefinite periods is unachievable, appropriate flame retardants (FRs) can turn these materials into hard-to-ignite materials, thus extending the range of their applications [15,16,17]. FRs are used to reduce the risk of fire in items by preventing ignition and delaying the spread of the fire and the flashover time, while also protecting the lifetime of the item and offering environmental protection by preventing local pollution and long-term environmental effects [18]. The most important parameters are a time to ignite, spontaneous ignition and flash point temperature, rate of heat release, thermal stability index, smoke toxicity, extinction flammability index, mass loss, limiting oxygen index (LOI), flame propagation on the surface, and fire resistance. Because of their high calorific capacity, polymers burn quickly. However, by adding FRs, it is possible to improve their fire behavior (e.g. by neutralizing or decreasing heat and smoke) [19]. Regarding these purposes, FRs have been introduced in the manufacturing of many goods to meet fire safety requirements.

Since the 1980s, there has been a growth in polymeric material utilization, which has enhanced the risk of fires caused by the flammability of polymeric materials [20]. To address this weakness, several FR treatments and techniques have been introduced, such as halogenated and non-halogenated FRs, layered silicates, nano fillers, copolymerization, grafting, and the synergistic use of natural fiber and FRs [21]. The two main categories of additive FRs are halogenated and non-halogenated refractory materials. Because they are inexpensive and effective, halogen-based compounds are the most used FR additions on the market. Several halogen compounds, however, have been banned due to their toxicity and environmental issues related to halogen-based refractory additives. The use of halogen-based compounds in the industrial sector of wood products in Europe has been prohibited since 2006 [22].

As a result, non-halogen refractory materials are becoming more widely used [23]. Environmental issues, mechanical/physical attributes, and processing constraints all necessitate a narrow range of options in the development of FR biocomposite materials. Nowadays, the need for unique FR solutions has been increased, with companies realizing the need for a product that is not only environmentally friendly but also long-lasting and cost-effective [18]. Polymer-based FRs are undergoing research and development. Because of their high availability and annual renewability, biobased FRs from animal origins, including chitin, DNA, and biomass sources (e.g., those that are cellulose based, such as lyocell fibre, saccharide based, and those based on polyphenolic compounds, etc.), hold promise in terms of their potential as “green” FRs in the development of biobased composites [24,25,26,27,28,29]. Aromatic compounds such as lignin and tannin are well known for their capability for producing char in combination with phosphorous [30]. Furthermore, the FRs employed must be safe for humans and animals, i.e., they must not emit hazardous compounds during normal material use. Using non-toxic nanofillers in polymers to achieve flame retardancy is a viable option [31]. Markedly, the addition of FRs in the matrix can result in the compromised physical and mechanical properties of the fabricated composites [18,19]. The aim of this research work was to present and discuss the recent advances in the development of fire-resistant biocomposites. The flammability of wood and natural fibers as material resources to produce biocomposites was evaluated to build a holistic picture. Furthermore, the potential of lignin as an eco-friendly and low-cost FR additive in the matrix of biocomposites with improved technological and fire properties was investigated. The limitations and perspectives of the economic and environmental elements of FRs were also highlighted for future implementation.

## 2. Flammability of Biocomposites

### 2.1. Woody Biomass

Biomass is the richest natural resource on the planet. Lignocellulosic biomass has gained increasing research interest because of its renewable nature [32]. Lignocellulosic biomass refers to both non-woody and wood biomass, which differ in their chemical and physical composition [33]. Holocellulose (a mixture of hemicellulose and cellulose) and lignin make up the category of lignocellulosic biomass. The composition of lignocellulose highly depends on its source, i.e., whether it is derived from woody or non-woody biomass [34,35]. Woody biomass is denser, stronger, and physically larger than non-woody. Furthermore, wood fibers can be collected throughout the year, minimizing the need for long-term storage [36].

One of the main disadvantages of wood as a structural material is its dimensional instability in conditions of environmental change. Wood is also susceptible to wood-destroying organisms such as insects and fungi, not to mention the fact that wood fibers are flammable. However, because wood has many advantages, such as excellent mechanical strength and insulation properties, and a pleasing appearance, it is commonly employed as a building material [37]. Furthermore, wood biomass is renewable and can be used as an organic matter-based sustainable energy source. A range of energy sources, including renewable energy sources, fossil fuels, solar energy, and nuclear power, can be utilized to generate electricity or other forms of power [38].

Several factors can influence the moisture content of wood biomass used as a source of energy [39]. In woody biomass, there are two forms of water, i.e., free water and bound water [40]. The cell cavity contains free water, whereas the cell walls of wood (cellulose and hemicellulose) contain bound water. Bound water, on the other hand, is repressed in wood’s chemical constituents, which contain hydroxyl groups that generate strong intermolecular hydrogen bonds. As a result, drying is necessary to lower the moisture content of wood [41]. The moisture content of wood biomass lowers its overall calorific value. 

Meanwhile, the anatomy of wood has an important impact on the pace of combustion [42]. Wood is mostly treated with FRs [43], which are usually inorganic salts, e.g., mono-diammonium phosphate, zinc chloride, ammonium sulfate, boric acid, sodium tetraborate, and other compounds. The use of refractory salt is applied to materials intended for interior applications only because it is not stable to washing with water [37]. Furthermore, some lignocellulosic plants have developed refractory behavior [30]. Due to their intrinsic capacity to form a thermally stable charred residue when engaging with fire, cellulose and lignin, which are the major constituents of lignocellulosic plants, have certain potential when it comes to their use as FRs additives [44].

**Figure 1:**
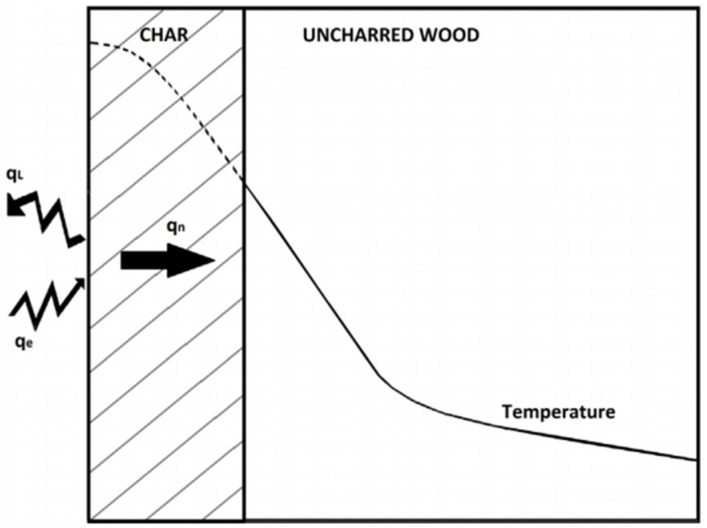
Heat transport in a charred wood sample [30]. Copyright @ 2017 Elsevier, License Number: 5157971091655.

Wood, due to its organic nature, is a combustible material. The burning rate of wood is determined by its density, air oxygen concentration, wood moisture content, and heat flux, and it is one of the most vital aspects of fire behavior [45]. The combustion rate refers to the rate at which a specific material is reacted by fire. It can be expressed in terms of mass loss, heat release, or char generation [46]. The process of heat transport in a charred wood sample is presented in Figure 1. When wood is subjected to heat, the surface temperature rises to the point where moisture content is removed, and the constituents of wood (lignin, cellulose, and hemicellulose) begin to decompose at a temperature of 160–180 °C. The pyrolysis and flame combustion of wood occur at temperatures greater than 225–275 °C. If given a spark, wood can burn at 350–360 °C, and the deterioration process begins with the development of a charred layer [30], while carbonization occurs within the range of 500–800 °C.

### 2.2. Non-Woody Biomass

**Figure 2:**
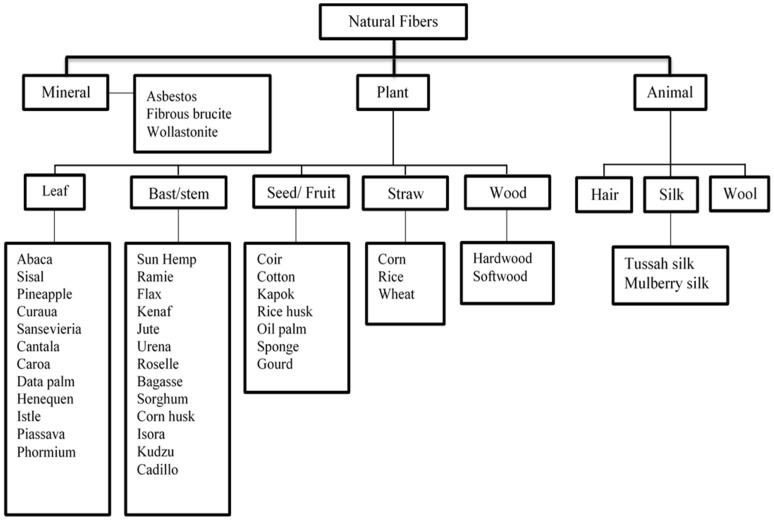
Classification of natural fibers modified from [48]. Copyright @ 2014 Elsevier, License Number: 5212860548284.

Non-wood fibers and wood fibers are the two types of natural fibers (Figure 2). Material obtained from agricultural waste or non-wood plant fibers is known as lignocellulosic biomass. The worldwide availability and biodegradability of lignocellulosic fibers, their low cost compared to synthetic fibers, and good mechanical properties, have resulted in increased industrial and scientific interest in the context of their wider utilization in the production of biocomposite materials. In addition, the use of lignocellulosic biomass has created new business development opportunities in countries with deficient fossil fuel stocks, which has provided conditions for sustainable development. Biomass obtained from crop residues on farmland or material leftovers after crops have been processed into usable goods is referred to as “agricultural residues”. Most agricultural waste is used as fertilizer or animal feed. Meanwhile, to save time and effort, some may be disposed of by burning or landfilling [47].

Non-wood biomass is a great potential raw material because of its better characteristics and endurance, as well as its ease of modification [49]. Textiles, paper, fabrics, biofuels, and composite reinforcing materials can all be made from natural fibers or non-wood plant species. In the automotive sector, composite reinforcement can be used for packaging, construction, and use [26,50]. Because non-wood fibers are more readily available than wood fibers, they are gaining increased attention as biomass feedstock for bioproducts. Non-wood fibers also have a more open structure, making them easier to process, which results in less processing energy. Furthermore, non-wood fibers are less expensive than wood fibers due to the fact that the majority of non-wood fibers are derived from perennial plants with a predictable supply [51].

**Figure 3:**
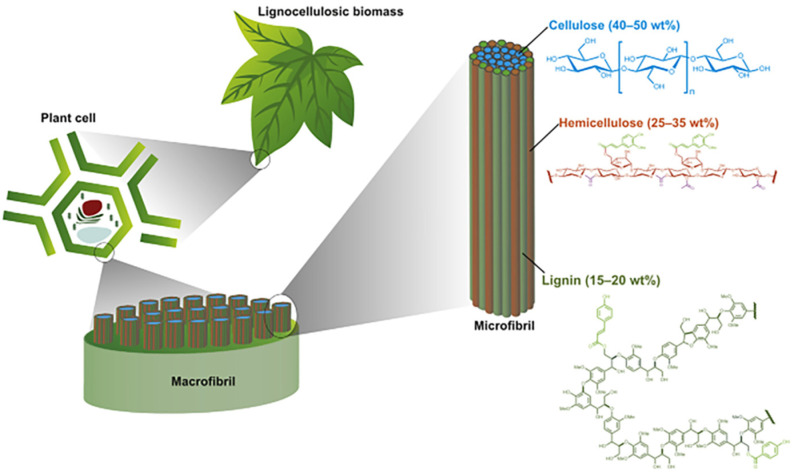
The cell wall structure of lignocellulosic biomass [62]. Copyright @ 2020 Elsevier, License number: 5157980402686.

Hemicellulose, cellulose, lignin, and pectin are all components of lignocellulosic biomass, which includes both non-wood and wood [52], with the proportion amount varying depending on plant species, tissue, growth stage [53], growth location [54], and axial position [55], as illustrated in Figure 3. Other constituents include extractives, ash, pectin, and waxes [56,57]. Plants are made up of several types of cells with varying physical properties, which are represented in proteins, structural components (polyphenolic compounds, and polysaccharides), and lipids. The presence of stiff cell walls with thicknesses varying from 0 to 10 µm in all plant cells determines their mechanical strength, their resistance to disease, while also influencing cell adhesion properties and the crucial interactions that allow plants to adapt to a variety of environments [58,59]. Natural fibers have a fiber diameter of 10–30 µm and are separated into three main layers: the outside primary cell wall, the inside secondary cell wall, and the outside secondary cell wall [60]. Plant cell walls can govern organ growth as well as the ability to withstand tensile or compressive stresses [59,61].

Cellulose fibers are hydrophilic, which means they absorb water. The moisture level of the fiber can range from 5% to 10%. This can result in dimensional variances in the composite, as well as a change in its mechanical characteristics. Hemicelluloses are responsible for fiber biodegradation, water absorption, and thermal deterioration, while lignin, which is thermally stable, is responsible for UV degradation. Lignin works as a natural adhesive, providing a protective barrier that prevents water and enzymes from accessing cellulose, increasing a plant’s resilience to pathogens and biomass breakdown. Some studies have summarized the variation of chemical components including lignin, hemicellulose, and cellulose in natural fiber [1]. Generally, fibers are made up of 40–60% cellulose, 10–25% lignin, and 20–40% hemicellulose [25]. Even though natural fibers have many advantages when it comes to the reinforcement of biocomposites, including annual renewability, lower production costs, good specific mechanical properties, reduced energy consumption during manufacturing, biodegradability, etc., their hydrophilic nature and poor fire resistance has become a limitation when it comes to expanding their range of uses [63]. Due to its low molecular weight, hemicellulose degrades quickly in the presence of heat. Lignin, meanwhile, has a unique highly aromatic structure and a high charring capacity upon heating at elevated temperatures, which decreases the heat release rate and combustion heat of polymeric materials, making it a feasible FR additive option [64]. Together with hemicellulose, lignin contributes to flame degradation properties [65].

The flame retardancy of natural fibers is primarily affected by their chemical composition, as well as their crystallinity and orientation. The characteristics of the resulting natural fiber reinforced composites (NFRCs) are affected by the fiber content, matrix types, filler concentration, compatibilizer, and fiber surface treatment [65].

At temperatures of 200–260 °C and 260–350 °C, respectively, hemicellulose and cellulose begin to degrade. During thermal decomposition, char, volatiles, and gases such as CO, ethylene, and methane are generated. Levoglucosan is generated at temperatures ranging from 280 to 350 °C. As the temperature rises, decomposition produces combustible volatiles, fumes, and carbonaceous char. Lignin is thermally degraded at temperatures of 160 to 400 °C. Bond cleavage takes place at lower temperatures, whereas aromatic ring bond cleavage takes place at higher temperatures [66]. Plant biomaterials have a high degree of biochemical and physical complexity due to the variety in the composition and varying numbers of structural constituents in plant cell walls of diverse species and tissues, which makes the physicochemical characterization of plant biomass difficult [53].

Due to their abundant availability, biomass chemicals hold promise in term of their potential as FRs in polymers. The chemical reaction of cellulose during heat degradation that results in char formation is exceedingly complex and perplexing, and is therefore disputed [36]. Natural fibers can be used as a fuel source, are susceptible to ignition and combustion, and are strongly consumed during combustion [63,67]. Natural fibers have a significant amounts of carbon, hydrogen, and oxygen, making them highly combustible [68]. They are an insulator with high mechanical qualities and a low thermal conductivity of 0.29–032 W/mK. Bark fibers are much less flammable than leaf fibers [68]. Increased thermal stability can be achieved by coating or adding chemicals [69]. The flammability of fibers is affected by their intermediate surroundings, which include the composition of the polymer matrix and other FRs present, the existence of a coupling agent, and the method used to produce the NFRCs. Horizontal and vertical burning tests, cone calorimeter testing, the LOI test, thermogravimetric analysis (TGA), differential scanning calorimetry (DSC), and dynamic mechanical analysis (DMA) have all been used to examine the flammability and thermal behavior of NFRCs [24,70,71,72].

Carbonization, followed by enhanced char production, is the mechanism of FR treatment of natural fibers [63]. Non-wood fibers are projected to play a larger part in the energy portfolio in the future, despite accounting for the bulk of biomass utilized in fuel generation [47]. Due to their thermoplastic and thermosetting properties, jute, sisal, coir, hemp, banana, bamboo, kenaf, sugarcane, flax, and a range of other natural fibers are used as a reinforcement alternative in polymer composites [64]. Due to their low lignin content, flax fibers have the highest thermal resistance among natural fibers, as measured by a long period before flashover and the duration to ignition. Meanwhile, jute fiber composites have the shortest duration but the fastest spreading fire with the least amount of smoke emission. The reduced smoke is a significant advantage because it diminishes the principal hazards of fire [73].

Vahabi et al. [24,28] have described a general mechanism for FR polymers in which they decompose with some activities in the condensed and/or phase phases, depending on the chemical composition of the polymer matrix and its chemical interaction with it. Modifying the decomposition pathway of polymers to create fewer combustible volatiles and more char, resulting in the production of a barrier or protective layer on the polymer’s surface, the cooling effect, and melt dripping are all achievable in the condensed phase. Some FRs aid in the production of polyaromatic structures and intermolecular processes during burning, resulting in carbonaceous char. The barrier effect is a well-known property of condensed phase solutions. In another piece of research, Nah et al. [74] have stated that FRs can act in both chemical and physical ways, e.g., by reducing flame spread, by raising the ignition temperature, by reducing the rate of burning, by cooling, and by forming a protective layer.

Some techniques, such as the chemical alteration of polymer matrices, have been used to provide flame retardancy. A phosphorus-containing reagent was used to chemically modify poly (vinyl alcohol) [75]. Aside from that, the FR coating of composites can be done in a variety of ways, such as using UV-curable boron in hybrid coatings or by using plasma coating techniques. Micro or nano FR incorporation in materials has also been reported to improve the flame retardancy and thermal properties of polymers [65]; however, the mechanical properties of the composites decreased [76]. To manage these qualities, suitable FR filler distribution, surface treatment, and compatibilizer addition are used [77,78].

### 2.3. Development of Lignin-Based FRs

**Figure 4:**
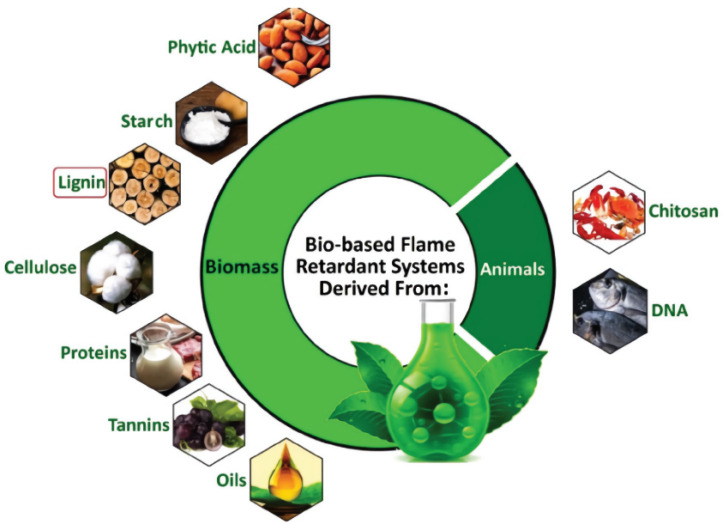
Schematic of various bio-based flame retardants from two main sources; biomass and animals. Copyright @ 2022 Elsevier Inc. All rights reserved.

Due to sustainability and environmental issues, the use of bio-based and renewable polymers and additives to improve fire retardancy has significantly evolved in recent years. There are two types of bio-based FRs: those that arise from biomass, such as lignin, starch, phytic acid, cellulose, tannins, proteins, and oils, and those obtained from animal DNA and chitosan [24,25], as presented in Figure 4. In recent years, lignin has attracted considerable attention in the context of promoting the FRs of polymers [79]. Lignin has a high thermal resistance, so it has great potential as an FR additive. It can also be effectively used as a carbon source for the design of intumescent systems in combination with other FR additives [80]. Numerous studies have demonstrated that using lignin or lignin derivatives can enhance the mechanical and thermal properties of polymeric materials [81]. The capacity of lignin to act as a flame retardant additive for polymers is highly dependent on its heat stability and ability to generate char [80]. The types and sources of lignin have a direct effect on their thermal decomposition behavior, which is usually characterized by a primary decomposition temperature range between 160–500 °C [82] and the fact that it produces a thermally stable product (char) at 700 °C [83]. The combination of starch or lignin with ammonium polyphosphate (APP) decreases LOI to an acceptable value (above 32%) [84]. Due to the fact that aromatic functional groups have varied thermal characteristics, observations regarding the thermal degradation of lignin cover a varied temperature range [85]. Table 1 summarizes a review of the literature on the development of lignin-based additives.

In the forced combustion test, the interaction between lignin and zinc phosphinates dramatically reduced the peak of heat release rate (PHRR), by 74%, and the total heat release (THR) by 22% in the mixture of lignosulfonate (LS) and kraft lignin (KL) [85]. Based on DSC and TGA test findings, KL impregnated with NH_4_H_2_PO_4_ and urea solutions raised the main degradation temperature (T_max_) from 541 °C to 620 °C and glass transition temperature (Tg) from 176 °C to 265 °C. The ignition time (T_i_) values increased by 339 °C, suggesting weaker thermal stability and fire resistance than KL itself [86]. After cone calorimetry testing, the combination of alkaline lignin with low sulfonic groups (LS) and ZnP in the polyamide matrix 11 (PA11) may reduce the PHRR value by approximately 50%, the THR value by about 13%, and the maximum value (MARHE) by 35% [87]. 

The chemical modifications of two lignins, kraft lignin (KL) and organosolv lignin (OL), by grafting phosphorus and nitrogen reduce the PHRR and THR by around 21% and 23% for KL with poly lactic acid (PLA) of 20%, respectively [88]. Functionalized lignin (F-lignin) with phosphorus-nitrogen grafting and a metal element (Cu^2+^) was used in the study of Liu et al. [90] to increase fire resistance and thermal stability. This reveals that PHRR values declined by 9%, THR values decreased by 25%, and average mass loss rate (AMLR) values reduced by 19%. Lignin can be added to the poly propylene (PP) matrix to act as an FR and toughening agent [90]. The use of alkali lignin (AL) in epoxy resins did not demonstrate a good FR in a study [91] since the carbon supply is solely in AL. The application of lignin as an FR in polymer composites is still falling short of industrial standards, such as its high LOI value > 28%. Traditional FRs such as APP, boric acid, and ammonium dihydrogen phosphate (ADP) can be employed as additives in the polymer matrix with lignin to achieve a high LOI while lowering the PHRR [93].

## 3. Challenges 

### 3.1. Fire Retardant Agents

Compounds containing halogens (i.e., bromine or chlorine), nitrogen, phosphorus, borax, boric acid, or inorganic metal compounds are often employed in refractory materials in the wood [94]. Ammonium chloride, ammonium sulfate, mono- and di-ammonium phosphate, boric acid, borax, calcium, zinc, aluminum chloride, and magnesium are among the key chemical constituents found in most FRs chemicals now on the market [94]. Coating, thermoplastics, thermosets, rubbers, and fabrics all have FR additive qualities that provide fire resistance. FR additives can prevent, minimize, and stop combustion, with the additive breaks the combustion cycle thereby reducing the burning rate of the fiber and, in cases, extinguishing the flame [95]. FRs are chemicals introduced to materials to decrease fires, increase thermal stability, control the spread of fires, or put a stop to combustion [96]. Table 2 shows the mechanism of action of FRs [97] when subjected to heat or when a fire occurs.

**Figure 5:**
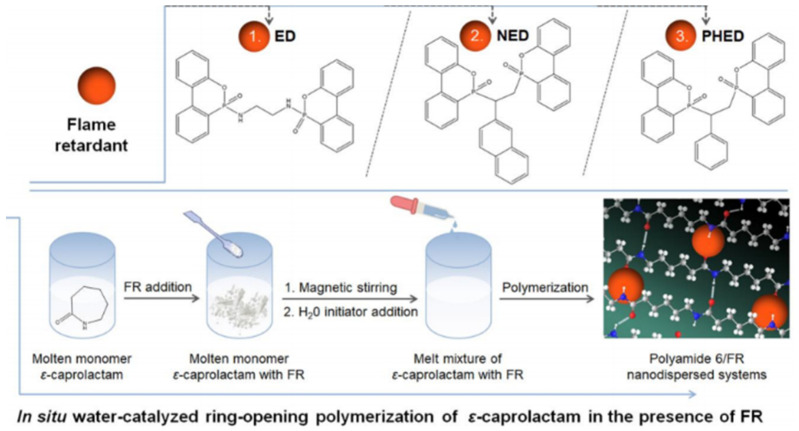
The fabrication method for the FR polyamide 6 (PA6)/FR nano-dispersed systems is based on the molecular structures of the flame retardants (FRs), i.e., ED, NED, and PHED [99] Copyright @ 2020 MDPI under CC by 4.0.

Due to its chemical makeup, polypropylene is readily flammable. Incorporating FRs into the polymer is one way to minimize its flammability. A neutralizing intumescent flame-retardant agent (NIFR) was produced and tested in polypropylene (PP) and found to be extremely effective as a fire retardant [98]. In situ liquid ring-opening polymers of both the ε caprolactam with a combination of ED (6,6′-(Ethane-1,2-diylbis(azanediyl)) bis(dibenzo[c,e][1,2]oxaphosphinine-6-oxide), NED (6,6′-(1-(2-Naphthyl)ethane-1,2-diyl)bis(dibenzo[c,e][1,2]-oxaphosphinine-6-oxide), and PHED (6,6′-(1-Phenylethane-1,2-diyl)bis(dibenzo[c,e][1,2]-oxaphosphinine-6-oxide) materials yielded the polyamide 6 (PA6) system, and the schematic for the preparation of PA6/FR nano-dispersed system can be seen in Figure 5. The advantage of using PA6 which is synthesized in situ from the three materials is that it has a uniform distribution, and the UL 94 test results for PHED material in the PA6 matrix show that it has an excellent fire-resistant effect that can decrease and prevent ignition [99]. 

**Figure 6:**
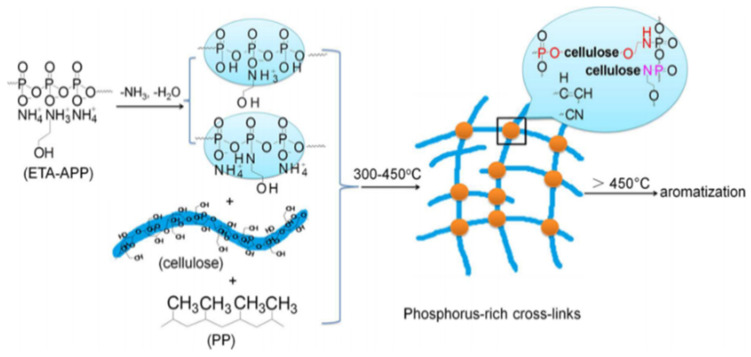
Pyrolysis process of WPC/ETA-APP biocomposites (30 wt.%) [100]. Copyright @ 2015 American Chemical Society under CC.

Wood flour in wood plastic composite (WPC) with 30% ethanolamine (ETA-APP) (modified ammonium polyphosphate) can provide fire-resistant characteristics while also increasing flexural properties. Figure 6 presents the probable creation method of WPC/30 wt.% ETA-APP. The ETA-APP produces phosphoric acid, which catalyzes cellulose chain scissions into many tiny molecules that may be destroyed quickly by heat. The release of NH_3_ and H_2_O results in the creation of stable molecules such as P-O-C, P-N-C, and CN, which react with hydroxyl cellulose to create a stable carbon layer and oxygen barrier. Polyoses, hemicelluloses, and lignin are all cross-linked [100]. 

**Figure 7:**
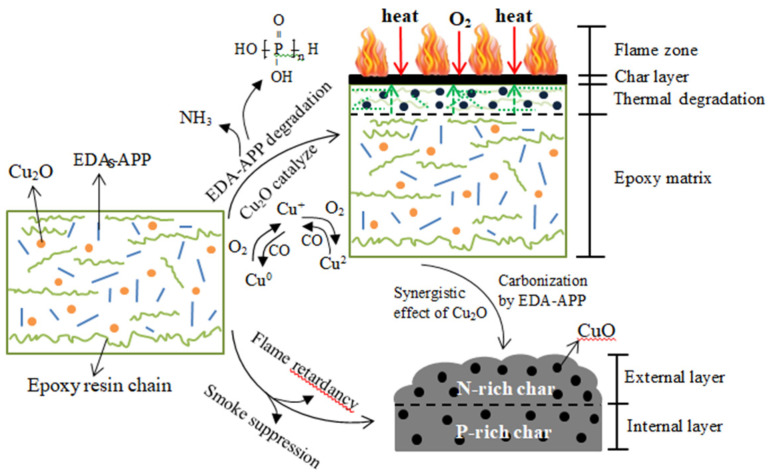
EP/EDA-APP/Cu_2_O composite’s potential flame-retardant and smoke-suppressant mechanisms. Modified from Chen et al. [102]. Copyright @ 2017 CC BY-NC 3.0.

In some reports, Cu_2_O acts as a synergist EDA-APP for epoxy/ethanediamine-modified ammonium polyphosphate (EP/EDA-APP) systems. Cu_2_O also acts as an adjuvant with EDA-APP in terms of increasing the quantity of char produced, intumescent degree, and compactness [101]. The EDA-APP may be utilized to make refractory neat EP composites. Cu_2_O can help to decrease smoke and carbon monoxide emissions. Figure 7 shows a schematic of the FR and suppression of smoke mechanism of EP/EDA-APP/Cu_2_O. Up to a temperature of 450 °C, phosphoric acids are formed and Cu_2_O hastens the breakdown of EP and EDA-APP, which can aid in the production of charcoal as a flammable barrier and reduce smoke and hazardous gas emissions [101].

The use of APP can be used as an FR; the material reacts with carbon compounds that can form charcoal as a protective layer which can prevent the further spread of fire [103]. Magnesium hydroxide (Mg (OH)_2_) is a chemical compound. It is also an FR substance which is capable of slowing the flame by releasing water at a temperature of 360 °C [104]. The addition of APP or APP combinated with zinc borate applied to sisal/polypropylene composites was able to increase fire resistance, thermal stability, and did not decrease their mechanical properties [105]. This coating technique can assist in increasing the composite’s fire resistance. It is applied during the finishing step or by impregnation [106]. Several chemicals, such as boron phosphate and silicon, have been found to improve the fire resistance of epoxy resin systems [107,108,109].

A method that is widely used in the context of adding active compounds to polymers is the addition of thermal (hydrated oxides) or inert fillers (silica, talc) to make less-flammable composite reinforced natural fiber [97]. The creation of several types of FRs from natural fiber reinforced polymer composites is depicted in Table 3.

### 3.2. Manufacturing FRs

Methods for preparing FR-treated natural fibers include the insertion of FR into adhesive and the mixing of fibers with FR before the addition of an adhesive [63] during the preparation process for biocomposites. Xiong et al. studied the development and application of refractory adhesives. In this research, a new design was studied to optimize the quality of fire-resistant adhesives as decorative panels for household needs. The orthogonal test determines the proportion of the adhesive with fire-resistant additives and the number of layers so that the fire-resistant adhesive can improve the performance of fire-resistant film-coated wood boards in the production process. The techniques of dyeing, peeling, strengthening the surface bond, and releasing formaldehyde follow national and industry standards. These techniques are also superior to the previous conventional technique, which used a panel wood-based veneer soaking technique by first using fire-resistant additives and then pasting them. The advanced technique can be used simultaneously with the production and treatment of refractory materials and their adhesives. Other advantages of this method are the emission of fewer materials used so that environmental pollution can be minimized, also the reduced use of FR additives and adhesives. Hence, the costs used are cheaper, and the production process is simple, easy, and eco-friendly [112].

Polybrominated diphenyl ethers (PBDEs) are materials originally utilized in consumer products such as foam furniture, padding, and electronics [113]. PBDEs are FRs that have been utilized in everyday products to prevent the spread of fire. They are added to numerous consumer products, including electrical circuits, building materials, thermoplastics, polyurethane foams, and other products as one of the most often used brominated flame retardant (BFR) classes [114].

As a result of the introduction of the flammability requirement, and the rising usage of synthetic materials, halogenated FRs have been in use since the 1940s, with a fast increase in demand and manufacturing since then. The growing demand has been satisfied by the development of new compounds with improved fire-resistant qualities [115]. PBDEs are one of the most frequently employed organic FRs, and they are found in a wide range of polymers used in construction products, consumer goods, and automobiles [116]. Furthermore, with the discontinuance of penta-, octa-, and decabrominated diphenyl ethers (BDEs), other “new” FRs (NFRs) were utilized in greater quantities to satisfy flammability regulations [117]. Table 4 lists a variety of commercial polymer refractory materials used in the fabrication of additives that meet the majority of fire safety criteria [23].

**Figure 8:**
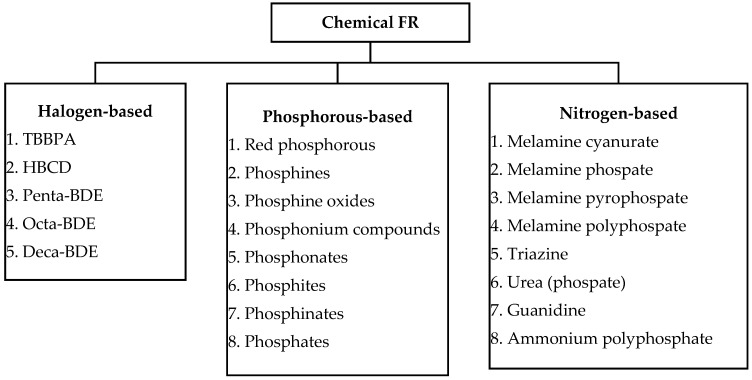
Flame-retardant additives. Modified from [23]. Copyright @ 2020 Elsevier, License number: 5157990641808.

Other thermally stable polymers can be employed in fire-prone situations, in addition to refractory materials. According to the flow chart in Figure 8, refractory additives (chemical FR) and materials with good heat stability are identified. There are three groups of chemical FRs, including halogen-based, phosphorous-based, and nitrogen-based FRs. Because flammability is not dependent on thermal stability, thermally stable polymers are not necessarily refractory [23].

### 3.3. Safety of FRs

Non-halogenated and halogenated FRs are the two types of FRs. Due to their durability, bioaccumulation, and potential human health impacts, halogenated flame retardants (HFRs) containing bromine or chlorine linked to carbon,] have gotten a lot of attention [96]. Halogen-based compounds are the most prevalent refractory additives on the market because they are cheap and effective. However, due to the environmental and toxicity problems connected with halogen-based refractory additives, several halogen compounds have been banned. The chemical interference with a radical chain mechanism in the gas phase during burning was used to prevent fires [118]. HFRs are used to lower the flammability of produced materials such as textiles, plastics, furniture, and polyurethane foam to inhibit the spread of fire [5].

Many home and commercial products contain FRs to minimize the fire consequences and losses, but the commonly used FRs, polybrominated diphenyl ethers (PBDEs), have negative consequences for human health and the environment [79]. To replace PBDEs, alternative flame retardants (AFRs) have been developed. The two primary components of AFRs, for example, are di-(2-Ethylhexyl)-tetrabromophthalate (TBPH) and 2-Ethylhexyl-2,3,4,5-tetrabromobenzoate (TBB), which is currently one of the most extensively used commercial fire-retardant mixes. A 1,2-bis (2,4,6-tribromophenoxy) ethane (TBE), pentabromobenzene (PBBZ), pentabromoethyl benzene (PBEB), and hexabromobenzene (HBB) are some of the other AFRs [117]. According to recent studies, the alternative level of FRs in the air has risen to levels comparable to PBDE [119]. Furthermore, due to their low cost, refractory additions to polymers containing halogen elements are frequently employed. Halogens, on the other hand, are reactive, as they can produce harmful and corrosive fumes. Due to their availability and low cost, phosphate-containing compounds such as phosphate are also utilized as an alternative to halogenated FRs. However, following further investigation, it was discovered that this refractory addition also possesses hazardous qualities [120].

At this time, a substitute for dangerous compounds in FR additives is required that performs similarly and may be employed in a safe and environmentally responsible manner [96]. The usage of polymeric composite materials that have not been treated with FR is hazardous to human safety [121]. Natural fiber/polymer composites are becoming more popular, and FR development must be evaluated in terms of human safety, human health, and environmental friendliness. Natural fiber has been more widely employed in the automobile industries and packaging [122], where fire safety regulations are less severe than in the aerospace industry. The flammability qualities and fire retardance function of biocomposites must be examined to widen their range of applicability for additional advanced manufacturing applications such as aerospace, electronics equipment, and building [63]. Several types of FRs are utilized in refractory goods, including aluminum, antimony, phosphorus, chlorides, bromides, and boron-containing compounds [123]. Metallic hydroxides, including magnesium hydroxide (Mg(OH)_2_) and aluminum hydroxide (Al(OH)_3_), and are commonly employed materials that are both safe for humans and environmentally benign [124]. Magnesium hydroxide (MgH), and aluminum hydroxide (ATH) were the refractory fillers utilized by Mohapatra et al. [120]. Non-halogen and non-phosphorous refractory additives were employed in this study. Inorganic fillers are becoming more important in industry due to their favorable mixture of low smoke, low cost, and reasonably good refractory efficiency.

## 4. Potential

### 4.1. Economic 

Fire is one of humanity’s greatest creations, but every year, approximately 4000 people in the United States and 5000 people in Europe pass away in fires and approximately 0.3% of the gross domestic product is lost as a result of fires [125]. In other countries, such as South Korea, the National Emergency Management Agency has reported a six-fold increase in large-scale fires (10 casualties, 5 lives lost, and $4 million in destruction of property) and in related damage (varying from 45 deaths to 232 deaths in 2019) and property costs (ranging from $5 million and $330 million) from 2010 to 2020. Mechanical and electrical problems are the primary causes of fires, contributing to large-scale fires [126,127], in addition to human error when ensuring the safety of electrical equipment in domestic buildings. Poor quality inexpensive electrical plugs and outlets are widely available in electrical stores and are primarily purchased by householders and small home industries due to their low cost [127]. During a fire, those who were near charred furniture may suffer burns, smoke, and poisonous gas inhalation, such as CO and other gasses (found in the soot in their airways), which may result in them becoming serious victims [128]. Aside from that, in several major cities in Indonesia, the construction of houses in densely populated areas is still largely based on wood [129] that vulnerable to fire. Residential settlements with very tight building distances make it easier for fire to spread and the type of flammable building (Regulation No. 14, 2012) affects fire vulnerability [127]. Throughout 2018, there were 698 cases of fires in Jakarta (DKI Jakarta Provincial Fire and Rescue Service, 2017) which counts as evidence for the high number of fires in Indonesia. Based on some data, the fire protection on the materials used in building construction, including furniture, becomes crucial even though it is very challenging to manage. The adverse effects of fire hazards can be mitigated by providing fire safety in buildings. The aim is to enhance fire safety through the establishment of rational fire design approaches, cost-effective fire removal technologies identifying innovative materials, defining performance codes, and evaluating wildfire fire hazard [130]. 

FRs, which are widely used additives in the plastics industry, are in high demand in the current global market. The market share of these agents is expected to be $2.3 billion [131]. Since the 1930s, halogenated FRs technology has been in use, demonstrating that FRs technology prices are quite low. Furthermore, boosting the fire resistance of composite materials could minimize the severity of incidents like aviation accidents [121], because the usage of composites without FR treatment is harmful to human safety. Typical biocomposites must be adjusted in several ways, such as chemical modification, to meet the high requirements and environmental demands of the circular economy. However, the higher flammability of biocomposites reinforced with lignocellulosic biomass when attacked by a heat flux or flame source can limit the wide uses of biocomposites. At 300–500 °C, cellulose-based polymers decompose into gas and condensed phases, resulting in incombustible liquids, char, gases, and smoke with potentially hazardous dripping. Therefore, some FRs have been introduced to decrease this negative effect associated with biocomposites’ easily flammable properties.

Nano-sized FR agents with a low or non-toxic environmental impact are increasingly used to improve the fire performance of combustible biocomposites [66]. They greatly limit the heat release rate, delay ignition, and slow flame propagation by adding 2–10% to the total material weight [121]. The challenge in developing FR biocomposites is how to maintain mechanical strength performance [132]. Furthermore, the usage of FRs raises the cost of the finished product [133]. Halogen-based FRs and nano-sized FRs have been known to advance the flame retardancy of composite even at lower concentrations compared to metal hydroxide compounds. However, because of their environmentally hazardous effect, their utilization is being banned. Intumescent FRs are new kinds of FR materials that considerably enhance flame retardancy, although further study is needed to improve this type of material [121].

### 4.2. Environmental

The flammability and post-ignition fire behavior of material and goods are increasingly regulated. The toxicity of FRs and FR-treated materials’ thermal composite products was not paid much attention in the context of them being heated, burned, or combusted for waste disposal, and there is a huge and growing tonnage of FR compounds in all stages of their life cycle. In recent years, toxicity issues concerning FRs have been a growing concern. One problem is that the small amounts of very dangerous combustibles that are emitted during unintentional fires and garbage incineration may cause environmental contamination. Further concerns relate to the occupational and environmental dangers in processing and recycling them, and technological problems in the recycling of particular materials [134].

Developing FR polymers with the potential to generate novel fire-safe materials is crucial. Some current FRs are subject to some regulations due to multiple environmental harmful effects. Despite the challenges, however, FR technology is progressing, and future fire-resistant polymer materials can reduce fire risks [126]. Globally, living standards and fire safety, including the prevention of ignition and flame propagation and extended escape times, are increasing because of the increased consumption of FRs [135]. Recently, the tendency has been towards them being more environmentally compatible. Concerning FR utilization, persistence, bioaccumulation, and toxicity (PBT), chemicals are a source of concern because they are degradation-resistant and can remain this way in the environment [136] and have been proven to be hazardous to humans and wildlife in recent years [137]. They have been known to be very effective and inexpensive for FR additives into the polymer. However, they pose toxicity issues during fires due to the formation of smoke, asphyxiants, irritants, and the direct discharge of irritating acid gases. Some environmental contaminations may have happened as a result of the emission of halogenated dioxins and dibenzofurans [134]. Halogenated FRs have greater levels of emission of corrosive fumes and gases during fires, possible environmental leaching, and are difficult to recycle [138]. Polymer composites and/or mixes have been used in some applications where fire danger and harm to humans and structures are key issues [63]. As a result, some efforts have been made to substitute them with more environmentally friendly FRs, such as non-halogenated FRs.

Melamine polyphosphate (MPP), a non-halogenated FR, has been demonstrated to be effective as an FR for bio-based thermoset polymer systems while having a minimal environmental impact in terms of toxicity [139]. Compounds that contain phosphorus are quite effective at preventing fire, are eco-friendly, and not very hazardous, especially when it comes to high-oxygen polymers [140]. Organophosphorus fire retardants are non-toxic and ecologically benign [141,142]. They have significantly lower toxicity than their organohalogen competitors, and may be made more effective by adding other components like sulfur, boron, nitrogen, or silicon [143]. In matrices which have an oxygen or nitrogen atom backbone, phosphorus and nitrogen-based chemicals are potential solutions when it comes to FR additions [65]. FRs containing nitrogen are one of the most environmentally friendly in terms of chemicals since they produce less smoke and produce no dioxins or halogen by-products during burning [137]. Nitrogen compounds are less effective than organohalogen (since phased out) or organophosphorus compounds at diluting the fuel load in the combustion zone by releasing inert fragments into the gas phase. Because nitrogen-based FRs decompose at a high temperature, they may be successfully integrated into thermoplastic polymers. When mixed with melamine phosphate and ammonium phosphate, they create a synergistic system [144]. Organophosphorus FRs derived from plants represent a promising solution for the development of biobased FRs due to their abundance, renewability, and non-toxicity. Isosorbide, a dihydroxy ether, is included in the category of organophosphorus FRs that can be produced from starch materials via the hydrolysis of glucose following the reduction of glucose followed by double dehydration [145]. It can also be extracted from seed grains [146]. By employing 5-hydoxymethyl-2-furfural (HMF) obtained from renewable resources, furan-based FRs constitute a harmless alternative to organophosphorus. Moreover, furan-based flame retardant (FBF) has a significant char yield and a high LOI [147].

When FR additives can be substituted by chemicals or systems which do not pollute the environment, while the fiber is bio accumulative inactive or toxicologically active, and when prices are comparable to the efficacy of FRs, FR additives can be advantageous [148]. The characteristic features and properties of several standard FR systems and products are summarized in Table 5 [148].

## 5. Conclusions

Increased environmental awareness, the scarcity of non-renewable resources, and recent technological advances have enhanced the industrial development of biobased composites with engineered properties for a wide range of value-added end uses. However, their flammability limits their broader use in more advanced applications. To improve fire protection in biocomposites, a wide variety of FR additives are commonly incorporated in their composition, enabling biocomposites with poor fire characteristics to fulfill regulatory fire performance criteria, widening their range of applications. This paper outlined the recent advancements in the development of fire-resistant biocomposites, providing an analysis of the flammability of woody and non-woody biocomposites. Due to its abundant availability and good thermal properties, the potential of lignin-based FRs in biocomposites as a ‘green’ alternative to the traditional FR compounds was also highlighted. Manufacturing biocomposites with FR properties, as well as their production properties and safety considerations, were described. Furthermore, the effects of incorporating FRs into biocomposites on the economy as well as their environmental impact were also presented and evaluated. Although it might be difficult to develop effective alternatives to the existing FR additives used in biocomposites for some applications, in most cases an improved fire performance can be obtained using biobased FRs with less environmental impact.

## Figures and Tables

**Table 1:** Development of lignin-based refractory additives from various biomass sources.

No	Method	Findings	Citation
1	Lignin in the form of LL and DL was combined with FR phosphinate (zinc phosphinates (ZnP) and aluminum phosphinate (AIP)) in Polyamide 11 (PA 11).	The mixtures PA80–LL7–ZnP13 and PA80–LL10–ZnP10 showed that these mixtures had the best fire resistance. Sulfonates were identified to play a role in the synthesis of thermally stable molecules in LL.	[85]
2	KL was soaked in a solution of ammonium dihydrogen phosphate (ADP) and urea in a ratio of 1:10 at 70 °C for 1 h.	Phosphorylated KL (PKL) can increase the value of Ti (ignition time), which is 399 ^°^C, which means that PKL has a superior thermal stability and FR qualities to kraft lignin	[86]
3	A mixture of LS and ZnP in a polyamide 11 (PA11) matrix was melted under pressure to form pellets and plates with a thickness of 3 mm.	A mixture of alkaline lignin with low sulfonate groups and ZnP can effectively improve the refractory properties of PA 11. Mixtures containing 10% alkaline lignin with low sulfonate groups and 10% ZnP showed the best performance.	[87]
4	Lignin was modified for phosphorus and nitrogen grafting. Melting procedure: a mixture of lignin and molten polylactic acid (PLA) was mixed at 160 °C for 7 min at 700 rpm per minute. Previously, before usage, the lignin and PLA were dried overnight in a vacuum oven at 60 °C (control PLA and composite PLA with 20% lignin type, i.e., KL and OL).	The thermal stability of OL is relatively small compared to KL in PLA-lignin composites because OL contains a lot of carboxylic acids and phenolic acid groups, which cause the high degradation of PLA during the melting process. The use of lignin as a refractory ingredient in PLA was proved to reduce the heat generated during combustion by forming a charcoal layer on the burned sample’s surface. The flammability of the PLA composite was lowered by lignin that had been changed by grafting phosphorus and nitrogen.	[88]
5	Alkaline lignin, ammonium polyphosphate (APP) and zein powder were tested. Lignin was dissolved in polyethylene glycol (PEG), then zein powder was added and mixed. Plastic was thermally molded (70 °C, 500 rpm for 10 min).	Lignin and APP additives can improve mechanical properties and refractory properties. By performing a vertical burning test and simultaneously degrading lignin, lignin can increase the FR characteristics of thermo-plasticized zein (TPZ). The best biocomposite is 10% APP and 3% Alkali lignin (AL), which has good tensile strength and stiffness	[89]
6	Functionalized lignin (F-Lignin) was created by dissolving 5.0 g lignin, 0.081 mL formaldehyde and 1.38 g DEP in 50 mL DMF following the addition of HCl, then put in 5 g of Cu (Ac)_2_ and washed and dried at 80 °C. Composite wood-polypropylene (Wood/PP) and wood-PP-lignin were made by melt compounding at 180 °C for 10 min at 60 rpm.	In the presence of FRs components (P and N), F-lignin is considerably better in improving the thermal stability and fire resistance of plastic composites, as well as the catalytic activity of Cu^2+^ on char production. F-lignin lowers the rate of smoke during combustion. The char residue increases and compact char is formed continuously during firing which is responsible for the increased fire resistance.	[90]
	Phenolization AL was made by mixing lignin alkali with H_2_SO_4_. Phenolated lignin (Ph-lignin) and melamine ammonium polyphosphate (MELAPP) were mixed to produce F-lignin-APP. FRs of EP composites were made by mixing F-lignin with an EP homogeneous, then adding diaminodiphenylmethan (DMM). A vacuum was applied until the resin is mixed.	F-lignin@APP applied to epoxy resins (EP) has strong fire resistance, while AL added to EP has less good fire resistance. F-lignin@APP can increase LOI values on EP, improve smoke suppression (9.9 m^2^) and lower the HRR (53.1 MJ/m^2^).	[91]
	Enzymatic hydrolysis lignin-based FR (Lig.) synthesis was modified with nitrogen and phosphorus. Lig was added to EP to make Lig/EP composites	Composite Lig-F/EP with high phosphorus content has the best fire resistance, and the ul-94 test results achieved an excellent V-0 smoke suppression rating with a significant reduction of THR, 46.6%, and smoke production, 53%. The increase in fire retardant quality was caused by the synergistic action of nitrogen and phosphorus.	[92]

**Table 2:** Important mechanisms of action on FRs.

Flame Retardants	Mechanism
Halogen-based flame retardant	The flame retardant mechanism is based on a radical reaction that acts on the vapor phase
Decabromodiphenly oxide, hexabromocyclododecane, and other bromine-based compounds	Bromine gases, FRs that frequently synergizes with antimony trioxide, can protect materials from oxygen and heat exposure while also limiting the chemical processes that occur during the condensed phase of combustion
Non-halogenated FRs	The char formation mechanism, acting on the flame inhibition of the condensed phase
Ammonium polyphosphate, sodium phosphate, and other phosphorus compounds	The material forms charcoal mainly as an automatic extinguisher by inhibiting oxygen contact and can protect against flammable gases gas
Compounds containing antimony, such as antimony trioxide	Capable of increasing charcoal production and scavenging free radicals through synergism with halogens
Metal hydroxide-based compounds, such as magnesium and aluminum	It works better as an insulator, slows the flame at high temperatures, and can dissolve smoke, making it safer for humans and the environment
Zinc borate, for example, is a boron-based chemical	Smoke output is reduced, and chars are produced
Melamine and its salts are nitrogen-based chemicals	This shows that phosphorus and nitrogen work together to generate char
Silicon-based compounds such as silica etc.	It provides a synergistic effect with APP by forming a layer of charcoal and silicon, also known as inert diluents

**Table 3:** Examples of fire-retardant natural fiber-reinforced polymer composites.

Polymers or Reinforcement Materials	Flame Retardants	Property Improvement	References
Wood fibers/PP composite	Silica and APP	APP and silica are excellent fire retardants for wood fiber/PP composite. Apart from tensile strength, the mechanical characteristics of the composites degraded after flame retardants were introduced.	[110]
Sisal/PP composites	Zinc borate and Mg (OH)_2_	The addition of FRs to sisal/PP can slow down the process while raising the temperature. The addition pf Mg (OH)_2_ and zinc borate to the sisal/PP composite can improve its fire resistance while not affecting its mechanical characteristics.	[77]
Cotton fabric/epoxy	Montmorillonite (MMT)	The thermal properties and flammability of the cotton fabric composite improved after treatment based on TGA study, vertical flame, and oxygen index analysis. There was no residue from the combustion on the control cloth, but on the MMT-treated cloth there was still some residue left.	[86]
Binder (flax short fibers/pea protein)	Some of the materials utilized in the manufacturing of aluminum tri-hydroxide include melamine phosphate (MMP), zinc borate (ZB), and melamine borate (MMB)	Using a protein binder, fire-resistant chemicals were integrated into insulating materials made from flax short fibers. MMB with 20 wt.% shows an increase in flame retardancy behavior.	[111]

**Table 4:** Flame-retardant polymers for additive manufacturing.

Name	Material	FR Additives	Machine
PA 2210 FR	PA 12	Phosphorus	P 385, P380i, P 380, P 360, P 350/2, P 700
PA 2241 FR	PA 12	Halogen	EOSINT P395/760/390/730
PA 606-FR	PA 12	Not identified	Unspecified
FR-106	PA 11	Not identified	Unspecified
DuraForm ProX FR 1200	PA 12	Not identified	ProX SLS 500
DuraForm FR 1200	PA 12	Unknown	sPro 60 HD-HS
DuraForm FR 100	Unspecified	Halogen free	3D systems
PEEK HP3	PEEK	-	EOSINT P800
ULTEM 9085	PEI	-	Fortus 400 mc/450 mc/900 mc
ULTEM 1010	PEI	-	Fortus 450 mc/900 mc
PPSF	PPSF/PPSU	-	Fortus 400 mc/900 mc

Note: Poly ether ether ketone (PEEK), polyphenylsulfone (PPSF), polyethylenimine (PEI): selective laser sintering (SLS), polyamide (nylon) (PA): polyetherimide (ULTEM): polyphenylsulfone (PPSU).

**Table 5:** Characteristic features and properties of common FR systems.

System	Possibility of Reducing Fire	Reduces Fire Size	FR System or Substance Bioavailability/Environmental Toxic Hazard	Toxic Combustion Product Yields	Toxic Product Yields in the Environment
Mineral wool, glass fiber, ceramic fiber, and aramid fiber are examples of inert insulation panels, layers, fillers, and interlinears.	Yes	Yes	Glass fiber and mineral wool are biodegradable if breathed in and have minimal absorption and toxicity. During installation or removal, there are certain minor health risks	There are not or not very harmful or toxic.	There are no or very minor environmental hazards
Coatings that are inflammable	Yes	Yes	There are no detected problems	No	None
Magnesium dihydroxide and aluminum trihydroxide	Yes	Yes, by releasing water.	There are no detected problems	No	None
Boric acid	Yes	Yes, due to the creation of glass.	REACH classification H360FD (may harm fertility): easily released from the substrate. Although there is a chance that the unborn child will be harmed, the risk of exposure is typically low. FR is regarded as a “green” product.	Reduced	Reduced
FRs of phosphorus, and nitrogen combined phosphorus	Yes	Yes, through char formation and in the gas phase.	It is contingent on the compound and the application’s durability	Some reduced	Reduced
Phosphorus halogen	Yes	Yes, primarily in the gas phase.	It is contingent on the compound, and the application’s durability	Enhanced	Enhanced
Ammonium polyphosphate	Yes	Yes, when it comes to char creation.	No detected issues	Reduced	Reduced
Organic halogens antimony salts	Yes	Yes, helps char in gaseous stages.	Some compounds have been phased out, depending on the chemical.	Enhanced	Enhanced
Inorganic halogens: PVC	Yes	To some degree	There is not any evidence that this is a problem (except VCM during manufacturing).	Enhanced	Enhanced
Fluoropolymers	Yes	Yes	It is unknown whether or not there is a problem.	Under some circumstances, such as HF and PFIB, there are increased severely toxic nanoparticles	Enhanced
Nanoparticle clays and fibers	Possibly	Possibly	Is it possible for nanoparticles to escape during use? What kind of toxicity happens?	There is a reduction in existing toxic compounds, but there is a risk of toxic exposure from aerosolized nanoparticles	Potential issue

Note: Vinyl chloride monomer (VMC), Registration, Evaluation, and Authorization of Chemicals (REACH), Perfluoroisobutylene (PFIB), Hydrogen Fluoride (HF).

## Data Availability

The data presented in this study are available on request from the corresponding author.
